# The Efficacy and Safety of Acupuncture for Treating Osteoporotic Vertebral Compression Fracture- (OVCF-) Induced Pain: A Systematic Review and Meta-Analysis of Randomized Clinical Trials

**DOI:** 10.1155/2021/8574621

**Published:** 2021-09-29

**Authors:** Jia-Liang Li, Sha Rong, Zhen Zhou, Xiao-Bo Zhang, Zhao-hui Tang, Qing-Song Huang, Wei-Hong Li

**Affiliations:** ^1^Basic Medical College, Chengdu University of Traditional Chinese Medicine, Chengdu 610075, China; ^2^People's Hospital of Shifang, Deyang 618000, China; ^3^Menzies Institute for Medical Research, University of Tasmania, Hobart, Tasmania, Australia; ^4^Hospital of Chengdu University of Traditional Chinese Medicine, Chengdu 610075, China

## Abstract

**Background:**

Osteoporotic vertebral compression fractures (OVCFs) are common health issues in the elderly that cause chronic pain in over one-third of patients. This study was sought to evaluate the efficacy and safety of acupuncture for alleviating pain caused by OVCFs.

**Methods:**

We performed a search of 8 electronic databases for publications from the inception to 30^th^ March 2021. Eligible studies were randomized clinical trials (RCTs) that evaluated the effect of acupuncture for the treatment of OVCFs. Two investigators evaluated literature quality and extracted data independently. RevMan V.5.4.1 was used for data analyses, with pooled risk estimates presented as mean difference (MD) or relative risk (RR) along with corresponding 95% confidence intervals (CIs), as appropriate.

**Results:**

Fourteen RCTs involving 1,130 patients were included in this meta-analysis. Compared with the control group, acupuncture showed a greater benefit on pain reduction caused by OVCFs (1 week: MD = −1.26, 95% CI: (−1.82,−0.70); 1 month: MD = −1.63, 95% CI: (−1.82,−1.43); 6 months: MD = −1.13, 95% CI: (−1.55, −0.70)). Acupuncture treatment was also associated with fewer adverse events, lower ODI index, and higher bone density than the control group (safety: (RR: 0.30, 95% CI: 0.12–0.75); ODI: MD = −3.19, 95% CI: (−5.20, −1.19); bone density: MD = 0.15, 95% CI: (0.05, 0.26)). The GRADE quality of these results was assessed as low or very low.

**Conclusions:**

Compared with the control treatment, acupuncture was more effective and safer in relieving the pain caused by OVCF and made a greater improvement on patient's ODI score and bone density. Given the low level of our study evidence, future high-quality studies are needed to verify our study findings.

## 1. Background

With population ages, the proportion of the elderly population (>60 years old) will continue to increase [1–3]. It is estimated that the proportion of the elderly population will reach 21.1% (approximately 2 billion) by 2050 [4–6]. Osteoporotic vertebral compression fracture (OVCF) is a common type of fracture in the elderly, with risk increasing continuously with age [7, 8]. Chronic pain is the major symptom of OVCF [9], which not only largely reduces patients' quality of life, but also imposes a heavy medical burden to aged healthcare [10, 11]. Analgesics are commonly used treatments in clinical practice for alleviating the pain symptoms of patients with OVCF, but there are still 40%–60% of patients who experienced intolerable pain within 2 years of the fracture [12]. Even for cured patients, their levels of pain are higher than the levels of their prefracture state [12]. Therefore, novel therapeutic approaches are required to further relieve pains and improve the patient's health [12].

Acupuncture is a traditional treatment approach in China that has been widely acknowledged worldwide [13]. Existing evidence showed that acupuncture is effective in treating most chronic pains, with and without being compared with other standard treatments [14–16]. Informed by it, the World Health Organization recommends use of acupuncture to treat a variety of pains including low back pain [13, 17]. In view of this, acupuncture has also been used by many clinicians for relieving pains caused by OVCF. However, there is lack of systematic evidence showing the effectiveness and safety of acupuncture on the management of pains caused by OVCF. To fill the evidence-practice gap and inform evidence-based clinical practice, we conducted this meta-analysis of randomized controlled trials assessing the clinical efficacy and safety of acupuncture versus controls in patients with OVCFs who underwent pain symptoms.

## 2. Methods

### 2.1. Data Sources and Selection Strategy

We searched published studies from the following electronic databases (4 Chinese and 4 English databases): PubMed, EMBASE, Cochrane Library, Web of Science, China BioMedical Literature (CBM), China National Knowledge Infrastructure (CNKI), Chinese Scientific Journals Database (VIP), and Wanfang database. Randomized controlled trials (RCTs) of acupuncture in the treatment of OVCF published from inception of the databases to 30 March 2021 were considered in the current study. The search was only limited to human studies, and no language restrictions were made. We used subject (“Osteoporosis” “Fractures, Compression” and “Acupuncture”) and free words (“Osteoporoses” “Bone Loss” “Bone Losses” “Compression Fracture” “Fracture, Compression” “Compression Fractures” “Acupuncture and moxibustion” “needle therapy” “needle” “Electroacupuncture”) jointly to search the titles and abstracts in the databases aforementioned. The search strategy was as follows, taking PubMed as an example:

“Osteoporosis”[Title/Abstract] OR “Osteoporoses”[Title/Abstract] OR “bone loss”[Title/Abstract] OR “bone losses”[Title/Abstract]
Fractures compression”[Title/Abstract] OR “compression fracture”[Title/Abstract] OR “fracture compression”[Title/Abstract] OR “compression fractures”[Title/Abstract]
Acupuncture”[Title/Abstract] OR (“Acupuncture”[Title/Abstract] AND “moxibustion”[Title/Abstract]) OR “needle therapy”[Title/Abstract] OR “needle”[Title/Abstract] OR “Electroacupuncture”[Title/Abstract]
(1) and (2) and (3)

Inclusion criteria: the following criteria were considered for the inclusion of RCTs in the present study (PICO format): (1) participants: No restrictions on country, race, language, age, and gender. Patients should be clearly diagnosed with OVCF by X-ray, CT or MRI examination, etc., with no established serious cardiovascular and cerebrovascular diseases and contraindications to acupuncture. Patients did not have cognitive impairment and pains caused by other diseases such as tumors and tuberculosis. (2) Intervention: acupuncture as the main treatment; a comparative study carried out between acupuncture and the control group. (3) Control: any type of control group including surgery, western medicine, etc. (4) Outcomes: Visual Analogue Scale (VAS, 0 point for painless and 10 points for extreme pain. Higher scores indicated more serious pains); Oswestry disability index (ODI); bone density and safety. (5) Study type: RCTs.

Exclusion criteria: studies with incomplete data and information, studies combined with other traditional Chinese methods. Also, duplicate articles were excluded.

### 2.2. Data Extraction

We used standard data extraction methods to extract data. The basic information, sample characteristics, intervention measures, outcome indicators, and other data, which were included in the article, were extracted by two investigators. In case of any inconsistency occurring in the result, this was further discussed by the two investigators or scrutinized by a third person.

### 2.3. Methodological Quality of Assessment

The quality of included studies was assessed by two investigators independently, using Cochrane risk assessment tool. Seven domains used for the quality assessment include: random sequence generation, allocation concealment, blinding of participants and personnel, blinding of outcome assessment, incomplete outcome data, selective reporting, and other bias. Each part can be graded into three categories: low risk, unclear risk, and high risk. Researchers made judgments based on the bias risk evaluation criteria of the Cochrane Handbook bias risk assessment tool.

The scoring system provided by The Grading of Recommendations Assessment, Development and Evaluation (GRADE) Working Group was used to assess the evidence quality for a specific outcome across studies. Five criteria were evaluated including study limitations, inconsistency, indirectness, imprecision, and publication bias. The evidence quality level of RCT is assigned an a priori as high but may be downgraded to intermediate, low-level, or very low-level if there is any identifiable bias.

### 2.4. Statistical Analysis

Data for the included literature was analyzed using RevMan 5.4.1 software downloaded from the Cochrane website. Data merging chose different methods according to the type of data. We chose the mean difference (MD) for continuous outcomes and risk ratio (RR) for categorical outcomes. Random effect model and fixed effects model were used to perform analysis, as appropriate. The *I*^2^ statistic was calculated to describe between-study heterogeneity. When *I*^2^ ≥ 50% and *P* < 0.05, we used the random effects model to pool the data; otherwise, the fixed effects model was selected for data merging. According to the different time lengths for VAS evaluation in each study, we formulated 3 different evaluation time length: 1 week (or 7 days), 1 month (4 weeks or 30 days), and 6 months. The ODI score and bone density measurement are based on the data collected after one month of acupuncture treatment. Subgroup analysis was performed according to different treatments combined with acupuncture (acupuncture combine with drugs, acupuncture combine with surgery). Sensitivity analysis was performed when necessary. Publication bias was estimated using a funnel plot (articles ≥10).

## 3. Results

### 3.1. Literature Screening

**Figure 1:**
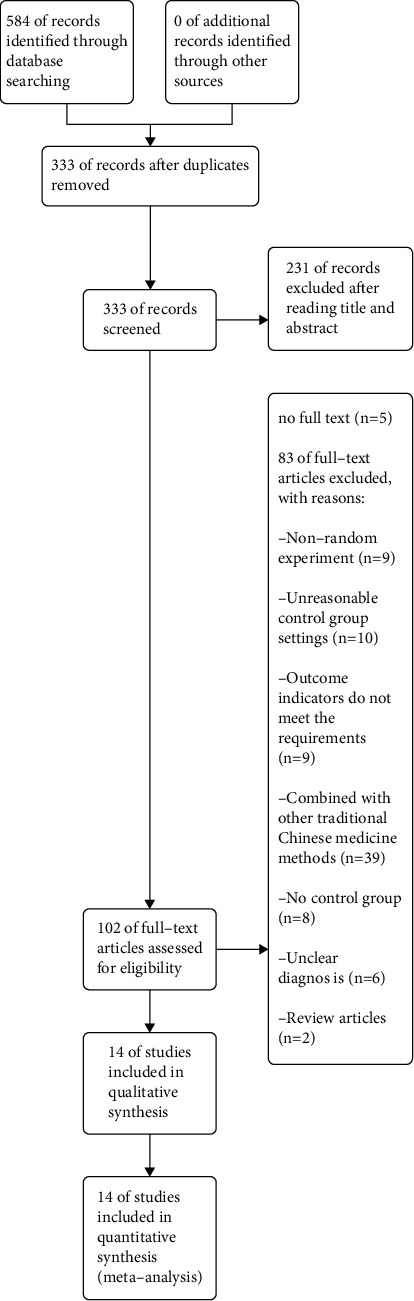
A flowchart showing the selection process.

We retrieved 584 articles initially. We then removed 251 duplicate articles manually leaving 333 articles. Out of 333 articles, 231 were excluded after reading title and abstract. Out of the remaining articles, five articles lack full text, and 83 were excluded because they failed to meet the inclusion criteria through complete reading. The present study eventually included 14 articles [18–31] (Figure 1), in which a total of 1,130 patients were randomly assigned to acupuncture-based (*n* = 573) or control therapy (*n* = 557).

### 3.2. Characteristics of Included Studies

Among the 14 (Table 1) RCTs that met the inclusion and exclusion criteria, 2 [25, 31] were in English, and 12 were in Chinese. Two [18, 27] were master's thesis, and the other twelve were journal articles. The study type of all included papers was single-center randomized controlled trial undertaken in China. Nine [18, 20, 23, 24, 26, 27, 29, 31] studies used surgery combined with acupuncture (including 4 percutaneous kyphoplasty (PKP) surgeries and 5 percutaneous vertebroplasty (PVP) surgeries). The intervention group received acupuncture analgesia after the operation, and the control group did not use acupuncture analgesia after the operation. Five studies used conservative treatment, the observation group of 4 studies [19, 21, 25, 28] used drugs combined with acupuncture treatment, and the control group only used drugs. Only one study [22], the observation group, was treated with acupuncture, and the control group was treated with medication. The shortest acupuncture intervention treatment time was 6 days, and the longest was 6 months. The intervention frequency of 11 studies was once a day, 1 study [26] every other day, 1 study [23] twice a week, and one study [18] based on different stages of the patient's disease course use of different frequencies (early stage (1 month after surgery): Qd in the first week; Qod in the second week; Biw in the third to fourth weeks. Mid-term (2 to 3 months after surgery): the first Week Qd; 2nd week Qod; 3rd to 6th week Biw. Late stage (3 to 6 months postoperatively): 1st to 4th week Biw). The observation time of outcome indicators (VAS) was not consistent across studies. The shortest VAS evaluation time was 48 hours, and the longest was 2 years. 2 [18, 23] out of 14 studies had an initial VAS evaluation time of less than one week, and 3 studies [18, 23, 30] had the last follow-up over 3 months, and the most time for outcome evaluation of VAS was 1 month after treatment. 8 studies [18, 20, 21, 24, 28–31] conducted VAS evaluation at this time. Only 3 [22, 25, 27] of 14 studies reported adverse reactions.

### 3.3. Methodological Quality

**Figure 2:**
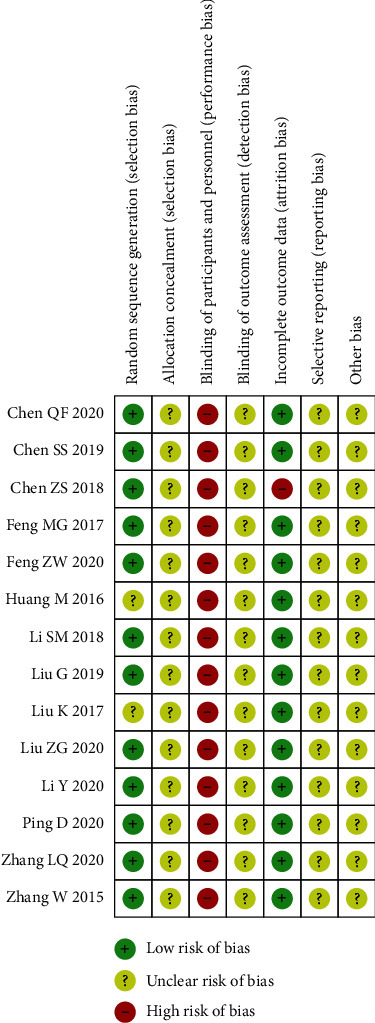
Risk of bias summary. +: low risk of bias; ?: unclear risk of bias; −: high risk of bias.

**Figure 3:**
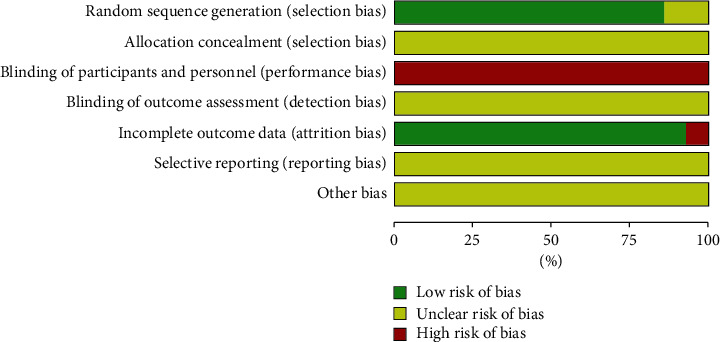
A graph showing the risk of bias.

The quality of the included studies is generally low (Figures 2 and 3). All studies were randomized controlled studies, 2 studies [18, 30] did not introduce specific randomization methods, 10 studies [19–21, 23–28, 31] used low-risk random number table grouping, 1 study [29] used lottery grouping, and 1 study [22] used the envelopes that were randomly grouped. No study used blinding for the allocation process. Due to the nature of acupuncture treatment, it was impossible to blind patients and practitioners in acupuncture-related RCTs. Among all the studies, the data of 13 studies were complete, and one study [23] reported missing data and was judged as high risk. Because no studies were registered in advance, it is not clear whether there is a risk of reporting bias and other risks.

We did not test the publication bias in this meta-analysis due to the insufficient number of studies for the analysis for each time period (<10).

### 3.4. Meta-Analysis of Effectiveness

A total of 1,130 patients were studied in the selected 14 studies, of which 573 in the acupuncture group and 557 patients in the control group. According to the different VAS evaluation times, we performed the evaluation at 5 time points.

**Figure 4:**
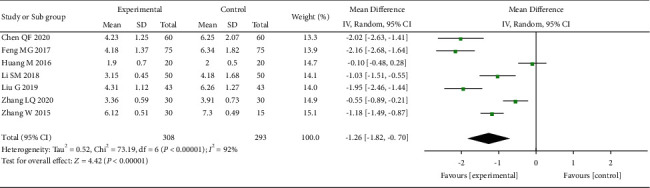
The forest plot shows the comparison of VAS between the acupuncture and control group in one week of treatment for pain caused by OVCF.

First, we evaluated the pain relief in a week after acupuncture treatment of OVCF (Figure 4), with 7 studies [20, 22, 24, 25, 27, 29, 30] of 601 patients included in the analysis. The combined results showed that the acupuncture-based treatment had a significantly greater efficacy in treating pains caused by OVCF than the control treatment (MD = −1.26, 95% CI: [−1.82 to −0.70], *I*^2^ = 92%).

**Figure 5:**
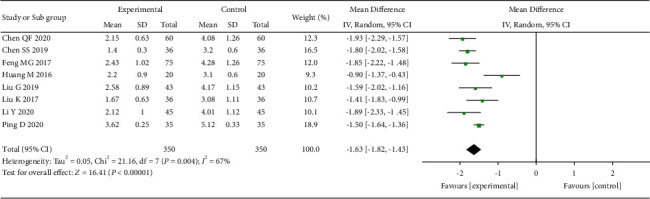
The forest plot shows the comparison of VAS between the acupuncture and control group in one month of treatment for pain caused by OVCF.

We evaluated the VAS after one month of acupuncture treatment for OVCF. A total of 8 studies [18, 20, 21, 24, 28–31] conducted VAS testing after one month of treatment. The heterogeneity among the studies was moderate (*I*^2^ = 67%). Random effects model was used to merge the data. The results suggest that the acupuncture has a greater efficacy than the control group (MD = −1.63, 95% CI: (−1.82, −1.43)) (Figure 5).

**Figure 6:**
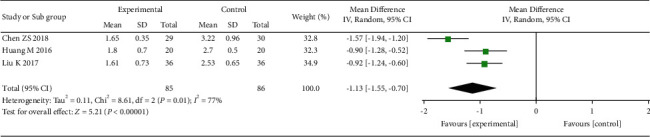
The forest plot shows the comparison of VAS between the acupuncture and control group in 6 months of treatment for pain caused by OVCF.

Finally, we evaluated the studies with a VAS evaluation time of 6 months **(**Figure 6**)**. There were three studies [18, 23, 30] that had the VAS evaluation time in 6 months, and between-study heterogeneity was large. The pooled results showed a greater analgesic efficacy of acupuncture treatment for treating pains caused by OVCF than the control group.

### 3.5. Safety Analysis

**Figure 7:**
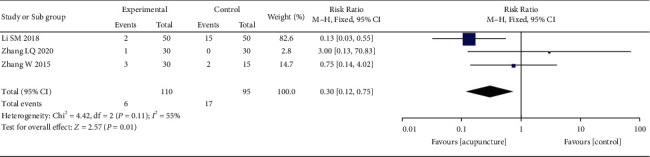
The forest plot shows the comparison between the acupuncture and control group in safety.

Three studies [22, 25, 27] reported adverse events. Compared with the control group, the risk of any adverse event was significantly lower in the acupuncture group (RR = 0.30, 95% CI: 0.12–0.75, *I*^2^ = 55%) (Figure 7).

### 3.6. ODI Score Analysis

**Figure 8:**
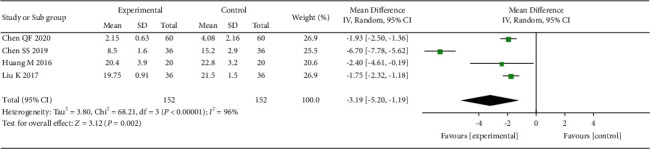
The forest plot shows the comparison between the acupuncture and control group in the ODI score.

Six of the 14 studies reported ODI scores of patients in different periods. We pooled the data during the period over which the ODI scores were collected most frequently (1 month). A total of 304 patients from 4 studies [18, 20, 30, 31] were included in this analysis. The analysis results show that, compared with the control group, acupuncture significantly reduces patients' ODI scores (Figure 8).

### 3.7. Bone Density Analysis

**Figure 9:**
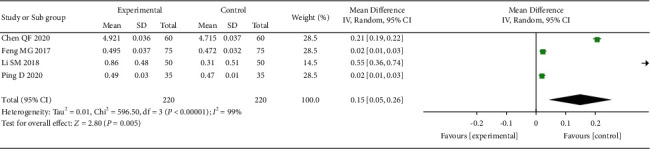
The forest plot shows the comparison between the acupuncture and control group in bone density.

Seven of the 14 studies measured the bone mineral density of patients. However, because of the inconsistency in the measurement method and the measurement time across studies, we only included 4 studies [20–22, 29] of 440 patients that used the same measurement method and the measurement time (1 month after acupuncture treatment). The results show that acupuncture significantly increased the bone density of patients compared with the control (Figure 9).

### 3.8. Subgroup Analysis

**Figure 10:**
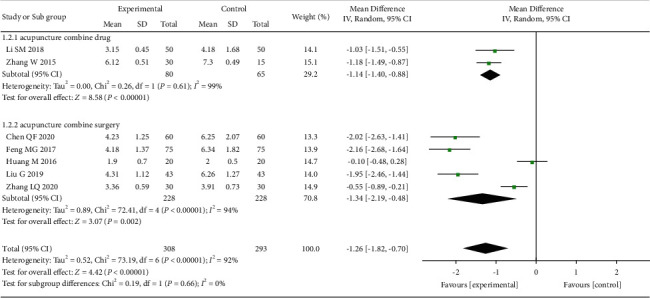
The forest plot shows the comparison of VAS between the acupuncture and control group in 1 week of treatment for pain caused by OVCF, subgroup analysis based on different treatment methods.

**Figure 11:**
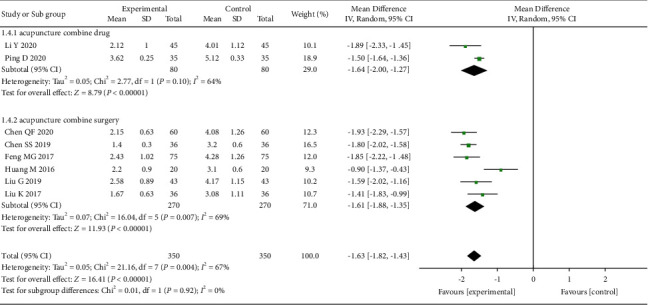
The forest plot shows the comparison of VAS between the acupuncture and control group in 1 month of treatment for pain caused by OVCF, subgroup analysis based on different treatment methods.

Subgroup analysis was conducted based on different treatment methods: acupuncture combined with surgery and acupuncture combined with drug (or only use acupuncture). Two time points were chosen to perform the subgroup analysis (1 week and 1 month). For both time points, combining the acupuncture with drug or with surgery was more effective in reducing pains caused by OVCF than the control treatment (Figures 10 and 11).

### 3.9. Sensitivity Analysis

**Figure 12:**
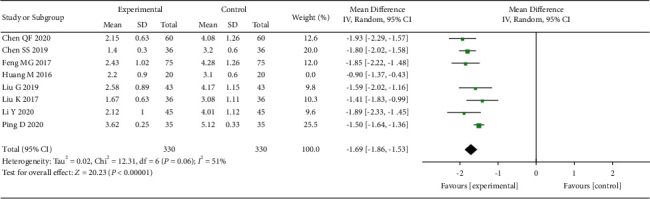
The forest plot shows the comparison of VAS between the acupuncture and control group in 1 month of treatment for pain caused by OVCF; one study is not calculated.

Given the high heterogeneity between the studies, a sensitivity analysis was performed at the time point of 1 month. Through the analysis, we found that one study [30] may be the heterogeneity source. After removing this study, the between-study heterogeneity was no longer statistically significant (Figure 12).

### 3.10. GRADE Evidence Quality Evaluation

The quality of evidence applied for each outcome was summarized in the ‘Summary of findings' table based on the GRADE approach (Table 2). The quality of evidence on the efficacy (one month), safety, the ODI score, and bone mineral density associated with acupuncture was rated as low, very low, very low, and very low, respectively.

## 4. Discussion

In this systematic review and meta-analysis, we found that acupuncture is better than the control group in treating pains caused by OVCFs over both short term and long term (from 1 week to 6 months), despite the large between-study heterogeneity for most outcomes. The pooled data from three studies reporting adverse events showed that acupuncture was safer than the control treatment in reducing pains caused by OVCFs. The study results also showed that, compared with control treatment, acupuncture significantly reduced the ODI score and increased the bone density of patients, suggesting a better clinical prognosis in those who received acupuncture. The subgroup analyses found that combining the acupuncture with either drugs or surgery was more effective than the control treatment in reducing pains caused by OVCFs. However, it is worth noting that the levels of evidence quality for outcomes ranged from low to very low.

By pooling the results from existing studies, our study found that acupuncture can effectively relieve pain caused by OVCFs, although the level of evidence was low. This study has several implications for patients with OVCFs. For patients who are treated conservatively, acupuncture can reduce the amount of use of analgesic drugs and the occurrence of side effects. For patients treated by surgery, acupuncture can promote earlier rehabilitation and improve clinical prognosis [18]. Although the mechanism of acupuncture alleviating pains is not fully explained, most studies suggested that it works by affecting the conduction of pain signals or by controlling inflammatory responses. Xu et al. [32] found that acupuncture could play a rapid analgesic role by inhibiting the upward conduction system of pain or promoting the downward inhibitory system and play an analgesic role in chronic pain by controlling the peripheral and central inflammatory responses. This finding is consistent with the research results of Lu et al. [17]. Liang et al. [33] and Wang et al. [34] studied the analgesic effect of acupuncture at the molecular level and found that the inactivation of spinal microglia and astrocytes mediated the immediate and long-term analgesic effects of electroacupuncture. Yang et al. [35] found that acupuncture can improve local blood circulation and promote the absorption of inflammation and edema, thus relieving pain at local sites. Abraham et al. [36] found that transient receptor potential vanilla-1 (TRPV1) may mediate the local analgesic effect of electroacupuncture. In addition to this, Chen et al. [37] showed that endogenous opioids, cholecystokinin octapeptide (CCK-8), 5-hydroxytryptamine (5-HT), noradrenalin, dopamine, glutamate, *γ* -aminobutyric acid (GABA), acetylcholine (ACTH), orexin A (OXA), and other substances mediate the analgesic effect of acupuncture.

Safety of acupuncture: our study concluded that the safety of acupuncture was higher than that of the control group. The favorable safety profile of acupuncture will promote more patients to choose it as a preferential treatment [15], especially those who are vulnerable and intolerable to the standard treatment. Although some patients experience fainting and bruising during the acupuncture, these adverse events are generally mild and faded away shortly after treatment. In this meta-analysis, only three studies reported adverse events related to study treatment assignment; thus, the reporting bias cannot be ruled out. More robust evidence is still needed to inform the safety of acupuncture in clinical practice.

Effect of acupuncture on ODI index: acupuncture can reduce the ODI index of OVCFs patients, a gold standard for the self-reported evaluation of the quality of life of patients with lumbar spine [18]. Since the pain is a major factor that affects the ODI index, the decreased ODI index with acupuncture is a likely result of its beneficial effect on pain relieving.

Effect of acupuncture on BONE mineral density: decreased bone mineral density is an important factor leading to OVCF in patients. Our study suggested that acupuncture could improve bone mineral density of OVCF patients, which was consistent with the research results of Wang et al. [38] and Zeng et al. [39]. Wang Gang et al. found that warm acupuncture at Back-Shu points and Jiaji (EX-B 2) points, the common points in OVCF, could reduce the BGP and IL-6 levels in serum, thus reducing bone absorption and inhibiting excessive bone turnover, and finally increasing bone mineral density and improving clinical symptoms. Through animal experiments, Wu et al. [40] found that acupuncture at Back-Shu points and Mingmen point could delay bone loss and improve bone strength and ultrastructure.

Factors influencing acupuncture: the effect of acupuncture can be affected by intervention frequency, number of acupuncture, acupuncture retention time, etc. Taking acupuncture retention time as an example, studying the time of acupuncture retention for tinnitus treatment, Wen et al. [41] found that the effective rate of acupuncture retention for 60 minutes was higher than that for 30 minutes, a timing used frequently in clinical practice. By studying the retention time of acupuncture in the treatment of perimenopausal syndrome, Yang et al. [42] found that the effective rate of electroacupuncture after 25 minutes was 100%, the effective rate of electroacupuncture after 40 minutes was 95.1%, and the effective rate was 92.31% when the needle was not retained (1–5 minutes). The timing of acupuncture plays a role on its effect. However, the benefit obtained does not simply increase with increasing timing. The times of acupuncture used also affect its efficacy. In our included literatures, there is a study [30] that the retention time of acupuncture was 20 minutes, and the times of acupuncture used were six; also, in this study, we found that it was the main source of heterogeneity when we conducted sensitivity analysis. Our analysis results showed that the commonly used acupoints were Back-Shu points and Jiaji (EX-B 2) points at the level of bone fracture, and the frequency was commonly once per day. Treatment duration was longer than 2 weeks in all but one study; retention time: 12 studies had retention time greater than 30 minutes (one was not recorded), and the longest retention time was 45 minutes.

### 4.1. Limitations

Several limitations of this study should be noted. First, all the included studies were single-center RCTs conducted in China; thus, our study results may not be generalizable to patients from other regions or countries. Second, except that the study itself is difficult to implement blinding, all studies lacked blinding of allocation methods and evaluators, and no studies were registered in advance before trial conducted. These might have largely reduced the quality of evidence generated from our analysis. Third, the heterogeneity between studies was large. Subgroup analysis and sensitivity analysis revealed that acupuncture combined with different treatment methods, retention time of acupuncture, and frequency of acupuncture were possible sources of heterogeneity. We only analyzed the heterogeneity at one point in time and did not analyze the heterogeneity in other time points because of the small number of studies. Fourth, the acupuncture points used in the studies included in this meta-analysis were different. This might have led to the differences in effectiveness of acupuncture between studies and contributed to the between-study heterogeneity. Last, the level of evidence for our findings was from low to very low. More high-quality studies are needed to validate our study results.

### 4.2. Implications for Future Research

This study suggests that acupuncture can be used as a useful supplementary approach adding to the drugs and surgery in alleviating the pain caused by OVCFs. Future studies with a more rigorous trial design are needed to improve trial quality, such as implementing allocation concealment to reduce selection bias. RCTs that are designed to compare the effectiveness of using different selected acupoints for acupuncture are also needed to facilitate the selection of the acupuncture points with greatest benefits for treatment OCVF-related pains. Given that all the studies included in this meta-analysis were conducted in China, relevant RCTs examining the effects of acupuncture on treating OVCFs-induced pains recruiting participants from other countries are needed. For the studies included in this meta-analysis, few evaluated the effect of acupuncture on the quality of life of patients. Future studies may target this area.

## 5. Conclusions

Compared with the control group, acupuncture is more effective in alleviating the pains caused by OVCFs over both short term and long term and has a favorable safety profile. At the same time, acupuncture can reduce the ODI score and increase the patient's bone density. Acupuncture combined with drugs and acupuncture combined with surgery are also more effective than simple drugs and surgery alone. However, given the low-level evidence quality, these results need to be interpreted with caution, and more high-quality studies are needed to provide more robust evidence.

## Figures and Tables

**Table 1:** Characteristics of included studies.

Author, year	Random method	Blinding	No. (acupuncture/control)	Age (years)	Acupuncture group			Control group	VAS evaluation time
					Intervention	Frequency	Treatment cycle	Intervention	
Zhang W, 2015	RNT	UC	30/15	55–80	M + A	QD	3 weeks	M	1, 2, 3 weeks
Liu K, 2017	UC	UC	36/36	≥65	PKP + A	Compound therapy	6 months	PKP	3 days, 1, 6, 12 months
Huang M, 2016	UC	UC	20/20	65–88	PKP + A	QD	6 days	PKP	1 week, 1, 6 months, 1, 2 years
Liu G, 2019	RNT	UC	43/43	57–77	PVP + A	QD	30 days	PVP	7 days, 30 days
Feng MG, 2017	Draw lots	UC	75/75	50–79	PVP + A	QD	30 days	PVP	7 days, 30 days
Chen QF, 2020	RNT	UC	60/60	52–79	PVP + A	QD	30 days	PVP	7 days, 30 days
Liu ZG, 2020	RNT	UC	43/43	60–71	PVP + A	Every other day	12 weeks	PVP	12 weeks
Chen SS, 2019	RNT	UC	36/36	50–65	PKP + A	QD	2 weeks	PKP	2 weeks, 1 month
Chen ZS, 2018	RNT	UC	32/31	48–80	PVP + A	BIW	6 months	PVP	2 days, 6 months
Zhang LQ, 2020	RNT	UC	30/30	50–85	PKP + A	QD	2 weeks	PKP	1,2 weeks
Feng ZW, 2020	RNT	UC	41/39	49–72	M + A	QD	6 months	M	3 months
Ping D, 2020	RNT	UC	35/35	60–80	M + A	QD	2 month	M	1 month
Li Y, 2020	RNT	UC	45/45	61–78	M + A	QD	3 months	M	30 days
Li SM, 2018	RE	UC	50/50	62–74	A	QD	2 months	M	7,14 days

M: medicine; A: acupuncture; RNT: random number table; RE: randomized envelope; UC: unclear; QD: Quaque die; BIW: twice a week; compound therapy, early stage (1 month after surgery): QD in the first week; Qod in the second week; BIW in the third to fourth weeks. Midterm (2 to 3 months after surgery): the first week QD; 2nd week Qod; 3rd to 6th week BIW. Late stage: 3 to 6 months postoperatively.

**Table 2:** Summary of findings.

Acupuncture for OVCF						
Patient or population: patients with OVCF						
Settings: inpatient						
Intervention: acupuncture						

Outcomes	Illustrative comparative risks^∗^ (95% CI)		Relative effect (95% CI)	No. of Participants (studies)	Quality of the evidence (GRADE)	Comments
	Assumed risk	Corresponding risk				
	Control	Acupuncture				

One-week VAS VAS Follow-up: mean 1 weeks		The mean 1-week VAS in the intervention groups was 1.26 lower (1.82 to 0.7 lower)		601 (7 studies)	⊕⊝⊝⊝ very low^1,2,3^	

One-month VAS VAS Follow-up: mean 1 months		The mean 1-month VAS in the intervention groups was 1.69 lower (1.86 to 1.53 lower)		660 (7 studies)	⊕⊕⊝⊝ low^1,3,4^	

Six-month VAS VAS Follow-up: mean 6 months		The mean 6-month VAS in the intervention groups was 1.13 lower (1.55 to 0.7 lower)		171 (3 studies)	⊕⊝⊝⊝ very low^1,2,3,5^	

Safety	Study population		RR 0.3 (0.12 to 0.75)	205 (3 studies)	⊕⊝⊝⊝ very low^1,3,5^	
	179 per 1000	54 per 1000 (21 to 134)				
	Moderate					
	133 per 1000	40 per 1000 (16 to 100)				

ODI Oswestry dysfunction index (ODI) Follow-up: mean 1 months		The mean ODI in the intervention groups was 3.19 lower (5.2 to 1.19 lower)		304 (4 studies)	⊕⊝⊝⊝ very low^1,2,3^	

Bone density X-ray determination of bone density Follow-up: mean 1 months		The mean bone density in the intervention groups was 0.15 higher (0.05 to 0.26 higher)		440 (4 studies)	⊕⊝⊝⊝ very low^1,2,3^	

^∗^The basis for the assumed risk (e.g., the median control group risk across studies) is provided. The corresponding risk (and its 95% confidence interval) is based on the assumed risk in the comparison group and the relative effect of the intervention (and its 95% CI). OVCF: osteoporotic vertebral compression fracture; CI: confidence interval; RR: risk ratio; VAS: visual analogue scale; ODI: Oswestry disability index; GRADE working group grades of evidence: high quality: further research is very unlikely to change our confidence in the estimate of effect. Moderate quality: further research is likely to have an important impact on our confidence in the estimate of effect and may change the estimate. Low quality: further research is very likely to have an important impact on our confidence in the estimate of effect and is likely to change the estimate. Very low quality: we are very uncertain about the estimate. ^1^No description of allocation concealment and blinding. ^2^The heterogeneity is large and cannot be explained. ^3^Publication bias is not tested, but there is a lot of dedication. ^4^The heterogeneity was explained after we performed sensitivity analysis. ^5^Sample size is too small (*n* ＜ 300).

## Data Availability

The table data used to support the findings of this study are included within the article. The figure data used to support the findings of this study are included within the figure files.
